# The NG2 Proteoglycan Protects Oligodendrocyte Precursor Cells against Oxidative Stress via Interaction with OMI/HtrA2

**DOI:** 10.1371/journal.pone.0137311

**Published:** 2015-09-04

**Authors:** Frank Maus, Dominik Sakry, Fabien Binamé, Khalad Karram, Krishnaraj Rajalingam, Colin Watts, Richard Heywood, Rejko Krüger, Judith Stegmüller, Hauke B. Werner, Klaus-Armin Nave, Eva-Maria Krämer-Albers, Jacqueline Trotter

**Affiliations:** 1 Department of Biology, Molecular Cell Biology, Johannes Gutenberg University, Mainz, Germany; 2 Research Center for Immune Therapy, Institute for Immunology, Johannes Gutenberg University of Mainz, Medical Center Mainz, Mainz, Germany; 3 Clinical and Experimental Neuroscience, Luxembourg Center for Systems Biomedicine, University of Luxembourg and Centre Hospitalier de Luxembourg, Luxembourg, Luxembourg; 4 Department of Neurodegenerative Diseases, Hertie-Institute for Clinical Brain Research, and German Center for Neurodegenerative Diseases (DZNE), University of Tübingen, Tübingen, Germany; 5 Max Planck Institute of Experimental Medicine, Department of Neurogenetics, Göttingen, Germany; 6 Institute for Molecular Medicine, University Medical Center of the Johannes-Gutenberg University, Mainz, Germany; 7 Cambridge University, Dept. Clinical Neurosciences, Division of Neurosurgery, Cambridge, United Kingdom; 8 Cellular and Molecular Neurobiology, Max Planck Institute of Experimental Medicine, Göttingen, Germany; Massachusetts General Hospital/Harvard Medical School, UNITED STATES

## Abstract

The NG2 proteoglycan is characteristically expressed by oligodendrocyte progenitor cells (OPC) and also by aggressive brain tumours highly resistant to chemo- and radiation therapy. Oligodendrocyte-lineage cells are particularly sensitive to stress resulting in cell death in white matter after hypoxic or ischemic insults of premature infants and destruction of OPC in some types of Multiple Sclerosis lesions. Here we show that the NG2 proteoglycan binds OMI/HtrA2, a mitochondrial serine protease which is released from damaged mitochondria into the cytosol in response to stress. In the cytosol, OMI/HtrA2 initiates apoptosis by proteolytic degradation of anti-apoptotic factors. OPC in which NG2 has been downregulated by siRNA, or OPC from the NG2-knockout mouse show an increased sensitivity to oxidative stress evidenced by increased cell death. The proapoptotic protease activity of OMI/HtrA2 in the cytosol can be reduced by the interaction with NG2. Human glioma expressing high levels of NG2 are less sensitive to oxidative stress than those with lower NG2 expression and reducing NG2 expression by siRNA increases cell death in response to oxidative stress. Binding of NG2 to OMI/HtrA2 may thus help protect cells against oxidative stress-induced cell death. This interaction is likely to contribute to the high chemo- and radioresistance of glioma.

## Introduction

Oligodendrocyte precursor cells (OPC) in the CNS are characterised by expression of Nerve-glial antigen 2 protein (NG2, also termed chondroitin sulfate proteoglycan 4 (CSPG4)), a type 1-transmembrane protein and chondroitin sulfate proteoglycan. [1,2]. OPC are sensitive to oxidative stress, as seen in white matter disease of the newborn, where premature human infants suffer hypoxic-ischemic insults and OPC are damaged, leading to long-term white matter damage [3,4]. In Multiple Sclerosis, oxidative stress in lesions may also result in OPC death [5,6]. Many aggressive gliomas also express NG2, including so-called tumour stem cells [7–11]. NG2 expression by gliomas appears to promote chemoresistance and protect against cell death [12] and may also encourage tumour invasion [13] as NG2 promotes migration [14]. Understanding the regulation of stress-induced cell death and a potential role of the NG2 protein here is therefore of clinical interest.

Activation of apoptosis can occur via two pathways. In the extrinsic pathway, apoptosis induction is regulated by activation of cell-surface death receptors such as TNF or Fas [15], and in the intrinsic pathway apoptosis is activated by proapoptotic proteins such as Cytochrome C, Smac/Diablo or OMI/HtrA2 released from mitochondria in response to cell damage [16]. The serine protease OMI/HtrA2 is localized in the mitochondrial intermembrane space (IMS). The protein is strongly conserved from bacteria to humans and it is thought that the OMI/HtrA2 protease plays a role in essential cellular processes by acting as a chaperone [17,18]. However, under conditions of cellular stress, OMI/HtrA2 is translocated from the IMS into the cell cytosol in response to increased permeability of the mitochondrial outer membrane. In the cytosol, OMI/HtrA2 binds to the inhibitors of apoptosis proteins (IAPs) and degrades them via the OMI/HtrA2 protease activity, resulting in caspase activation and induction of apoptosis [19,20]. OMI/HtrA2 can also induce apoptosis in a caspase-independent fashion by degradation of anti-apoptotic factors via its protease activity [18,21]. The binding of ligands to the PDZ-domain can regulate OMI/HtrA2 protease activity [22].

Here we report that expression of NG2 has a protective effect in OPC under oxidative stress conditions through binding and thus sequestering OMI/HtrA2. This interaction reduces the protease activity of OMI/HtrA2. Furthermore, human glioma cells expressing high levels of NG2 are more resistant to induction of cell death by oxidative stress: reduction of NG2 levels by siRNA decreases their resistance. Expression of NG2 by OPC may thus aid in protecting OPC against induction of cell death by oxidative stress. In glioma cells, the interaction is likely to contribute to resistance to chemo- and radiation therapy.

## Materials and Methods

### Ethics Statement

Experiments were in compliance with the animal policies of the University of Mainz, approved by the German Federal State of Rheinland Pfalz, in accordance with the European Community Council Directive of November 24, 1986 (86_609_EEC). All animal experiments were carried out in strict accordance with protocols approved by local Animal Care and Use Committee of the Johannes Gutenberg University of Mainz. Mice were sacrificed by decapitation to remove the brain.

All human tissue materials (glioblastoma cells R10) were obtained at Cambridge University, Dept. Clinical Neurosciences, Division of Neurosurgery. Tissue collection protocols were compliant with the UK Human Tissue Act 2004 (HTA Licence ref 12315). This study was approved by the Local Regional Ethics Committee (LREC ref04/Q0108/60) and also approved by the central biosciences committee for animal research. Informed written consent was obtained from each patient through the research clinic [23].

### Antibodies and expression vectors

Antibodies used were as follows: rabbit anti-cleaved-caspase-3, rabbit anti-Poly (ADP-ribose) polymerase (PARP) (Cell Signaling Technology); mouse anti-β-tubulin isotype III, mouse anti-cyclic nucleotide phosphodiesterase (CNPase), mouse anti-FLAG, rabbit anti-Junctional Adhesion Molecule A (JAM-A) (Sigma-Aldrich); mouse anti-Cyclooxygenase-1 (COX1) (Invitrogen); rabbit anti-GFP which cross-reacts with EYFP (Abcam); rabbit anti-GFAP (Dako Cytomation); mouse anti-penta-HIS (Qiagen); mouse anti-gamma-enolase (Santa Cruz); rabbit anti-Aspartoacylase (ASPA) (kind gift from Dr. M. Klugmann, Sydney, Australia); rat monoclonal anti-NG2, which reacts with an extracellular epitope in the region between aa1237 and 1531 of murine NG2 [24,25], rabbit antibody Ki67 (Becton Dickinson). Secondary antibodies were purchased from Dianova and Invitrogen. Expression vectors used were as follows: OMI/HtrA2-FLAG was cloned into the EcoRV/Xhol site of the pCMV-Tag4 vector (Stratagene) (Strauss et al. 2005). cDNA from NG2 (with a deletion in the extracellular domain, Chatterjee et al., 2008) was amplified and cloned into the Nhel I/BamHI site of the pIRESshyg2 vector (Clontech) to obtain an NG2del-FLAG expression vector. In the NG2del- sequence the PDZ-binding motif is deleted (6975–6984), in the NG2del+ sequence the motif was intact. NG2del fusion proteins were also generated containing a HIS-Tag in place of the FLAG-Tag [14,26], thus allowing purification of the proteins using a nickel column.

### Yeast Two Hybrid Screen

The yeast two-hybrid system was used to map the PDZ-binding-motif at the COOH terminus of NG2. The entire 76-amino acid C-terminal region of mouse NG2 (NH2-RKRNKT. NGQYWV-COOH, GenBank accession numberAF352400) was fused to the Gal4 binding domain by cloning it into the pGBT9 vector (Clontech) with XbaI/HindIII. The resulting bait construct was designated pGBT9cyto. Using the lithium acetate method, the yeast strain CG1945 was transformed sequentially with pGBT9cyto and a 9–12-week-old postnatal mousebrain MATCHMAKER cDNA library in pACT2 (Clontech).

Individual mutations of the 0, −1, −2, and −3 positions of the COOH-terminal peptide QYWV* were introduced by PCR, cloned into pGBT9, and designated as NG2 0G (Val mutated to Gly), NG2 −1G (Trp mutated to Gly), NG2 −2G (Tyr mutated to Gly), NG2 −2F (Tyr mutated to Phe), NG2 −3G (Gln mutated to Gly). Mutant NG2 constructs were cotransformed with mouse OMI/HtrA2. Yeast cells were grown on double dropout medium and assayed for β-galactosidase gene activity and additionally selected for growth on triple dropout medium [27].

### Cell culture

The cell line Oli-neu was cultured in modified Sato media [28]. Cell culture dishes were coated with poly-L-lysine (Sigma). HEK293T cells were cultured in DMEM (Sigma) with 10% Horse serum (Biochrom) and 1% Sodium-pyruvate (Sigma). Transfection of HEK293T cells with the NG2del constructs was effected by a standard protocol using the GenePulserXcell (Bio-Rad). Transcription was increased by including 4 mM sodium butyrate for the protease assay.

Cerebella of postnatal day 8–9 homozygous NG2-EYFP(NG2-KO) mice [29] or C57BL/6N mice (as control) were dissociated in 1% trypsin, 0.05% DNase in HBSS using a fire-polished Pasteur pipette to obtain a single-cell suspension, followed by seeding on poly-l-lysine-coated dishes. The cells were cultured in B27 medium containing DMEM, pyruvate, triiodo-L-thyronine, L-thyroxine (Sigma), B27 supplement (Gibco), 10ng/ml PDGF, 5ng/ml FGF (PrepoTech) and 1% HS. The medium was changed on the following day and renewed every 3–4 d. After 10-14d (after morphological assessment) cultures were stressed with H_2_O_2_ in B27 medium without growth factors.

Glioblastoma cells (R10) were cultured on ECM (Sigma) gel-coated dishes. ECM Gel was diluted 1/10 with Neurobasal media. Growth medium was Neurobasal medium (Invitrogen) with N2 supplement, B27 supplement, L-glutamine, EGF, FGF and 1% [v/v] penicillin/streptomycin (Serva).

### Immunocytochemistry

Cerebellum cultures or Oli-neu cells were fixed in 4% paraformaldehyde in PBS and permeabilised with 0.1% (v/v) Triton X100 in PBS. After blocking with 10% horse serum in PBS, cells were incubated for 45min with primary antibodies followed by dye-conjugated secondary antibodies (30min) and were finally imbedded with Moviol. In some cases, nuclei were stained with DAPI. Images were acquired with Leica DM-6000 Deconvolution Fluorescence Microscope (20x or 40×/0.7 NA objective lens). Images were adjusted using Photoshop (Adobe) and analysed with ImageJ.

### Cell Lysates and Immunoprecipitation

Cells were scraped off in cold lysis buffer (50 mM Tris, pH 7.4; 150 mMNaCl, pH 7.4; 1% (v/v) Triton X-100; in some cases plus 30 mM n-octyl-glucoside) containing protease inhibitor mixtures (Roche) and incubated on a rotating wheel for 45 min. Postnuclear supernatants were obtained by pelleting the nuclei for 10 min at 1000 × *g* at 4°C.

For IP, 100μl packed protein A- or G-Sepharose were incubated with antibody solution (1–2 μg) for 2h. The Sepharose-beads were then incubated with lysates (overnight at 4°C). Sepharose without bound antibody served as a control (preclear). Beads were washed four times with 1 ml of lysis buffer and once with 1 ml of RIPA buffer. For precipitation of NG2, CNBr-activated Sepharose (GE-Healthcare) was covalently coupled with mcNG2 antibody according to the manufacturer's instructions.

### siRNA-based downregulation of NG2

Small interfering RNA (siRNA) transfection of Oli-neu cells and glioblastoma cells was performed using Amaxa Biosystems technology according to the manufacturer's instructions (Amaxa Nucleofector kit, Primary Neurons; program O-005). The siRNA against NG2 (target sequence in the UTR1) and the control-siRNA (target sequence: 5′-AAT TCT CCG AAC GTG TCA CGT-3′) were obtained from QIAGEN. Experiments were conducted 30 hours after transfection.

### Cell lines with stable knockdown of NG2

Oli-neu cells stabily expressing shRNA sequences directed against firefly luciferase (shLuc) as a control or against NG2 (shNG2, knock-down) as described in Binamé et al. [14] were used. Cell populations were selected with 4μg/ml puromycin for 3 days. The cells were then cultured on PLL coated 11mm coverslips (80,000 cells/coverslip) and fixed after 24h and 48h with 4% PFA for immunofluorescent staining (S1 Fig).

### CoIP-ELISA

NG2 antibodies were bound to 96 well plates (Nunc) overnight at 4°C. Blocking with 3% [w/v] BSA, 0,2% [w/v] gelatine was for 2h. Oli-neu cells were cultured for 20h and incubated with H_2_O_2_ for 4h. Then the cells were lysed with cold lysis buffer (50 mM Tris, pH 7.4; 150 mMNaCl, pH 7.4; 1% (v/v) Triton X-100; 30 mM n-octyl-glucoside) containing protease mixtures (Roche). After washing, incubation was performed with pcOMI/HtrA2 antibody (1:4000 in PBST) followed by incubation with HRP antibody and subsequently addition of TBM-substrate (Pierce). The reaction was stopped with 2M H_2_SO_4_ and converted TBM substrate was measured at 492 nm with a Plate Reader.

### Mito-Capture staining

The membrane potential of mitochondria was detected by Mito-Capture staining (Mitochondrial apoptosis Kit; PromoKine) according to the manufacturer’s protocol. Oli-neu cells were plated on 11 mm coverslips (10.000cells/coverslip) and stressed for 5 hours with different concentrations of H_2_O_2_. The monomeric form of the dye was acquired by (Ex/Em: 488/530nm) and the aggregated form by (Ex/Em: 488/590nm).

### H_2_O_2_ or ONOO^-^ Treatment and viability test (MTT-assay)

Cerebellum cultures were stressed with 800 μM H_2_O_2_ (Roth) for 18h without a media change (in order to obtain a dose-response curve), with the exception of the cultures used for measurement of cleaved-caspase-3: these were stressed for 5 hours. In some experiments 7.5 μM UCF101 was added 30min before stress treatment without a media change. Oli-neu cells, HEK293T or GBM (R10) cells were incubated with H_2_O_2_ or ONOO^-^ (Sigma) for 4–5 hours. Cell viability was assessed by the MTT assay [30]: 0,5 mg/ml 3-(4,5-dimethylthiazol-2-yl)-2,5-diphenyltetrazolium bromide (MTT, Sigma) in PBS was added to the medium for 2 h. Formazan crystals formed were solubilised in a buffer containing 40% [v/v] dimethyl-formamide (Sigma), 10% [w/v] SDS, and 2% [v/v] acetic acid overnight. The absorbance was measured at 562 nm using a plate reader (Amersham Bioscience).

### Measurement of LDH release

Oli-neu cells or glioblastoma cells (R10) were incubated with H_2_O_2_ or ONOO^-^ for 5 hours. After stress media was collected and the LDH release was assayed using an LDH cytotoxicity-detection-kit (Roche) according to the manufacturer’s instructions. The LDH-release was determined by measurement of the absorbance with an ELISA reader (absorbance _492 nm_- absorbance_600 nm_) and compared to unstressed controls.

### Western Blot analysis

SDS-PAGE analysis was performed using self-made gels or 4–12% NuPAGE gels (Invitrogen), according to the manufacturer's instructions. Proteins were blotted onto a PVDF membrane. The membranes were blocked for 30 min with 4% (w/v) milk in TBST (0.05 M Tris, 0.15 M NaCl, pH 7.2, 1% (v/v) Tween 20). Primary antibodies were incubated overnight at 4°C and secondary antibodies for 30 min at room temperature, all in blocking medium. Detection of primary antibodies was realised by secondary anti-species antibodies conjugated to horseradish peroxidase (HRP). The blots were developed with enhanced chemiluminescence reagents (Pierce) according to the manufacturer's instructions. Densitometric quantification of films was performed with ImageJ. For quantification of WB signals, samples were compared that were blotted on the same membrane.

### Protease Assay with β-casein

HEK293T cells were transfected with NG2del-HIS constructs and the cells were cultured with 4 mM sodium butyrate for 24h. Purification of the NG2del-HIS fusion proteins from the cell lysates was carried out using HIS-Select-affinity gel (Sigma) according to the manufacturer's instructions. For the protease assay the digestion of the OMI/HtrA2 substrate β-casein, as a generic substrate of OMI/HtrA2, was analysed [31,32]. HEK293T cells were transfected with OMI/HtrA2-FLAG. OMI/HtrA2-FLAG fusion proteins were isolated by IP with FLAG antibodies coupled to Sepharose beads. The OMI/HtrA2-FLAG IPs were then incubated with 15μg purified NG2del-HIS fusion proteins overnight at 4°C. Incubation with the elution buffer of the HIS-select affinity gel served as a control. After several washing steps, the OMI/HtrA2-FLAG IPs were incubated with 0.09 μg/μl β-casein in PBS for 5h at 37°C. The supernatants were analysed for casein digestion via gel electrophoresis and silver staining (Invitrogen). IPs were analysed by Western-Blot.

### Statistical analysis

For all studies significance was calculated using the Student’s t-test (paired or unpaired, two tailed: * = p<0.05; ** = p<0.01; *** = p<0.001). Values without asterisks are not significant. Error bars reflect standard error of mean (SEM) and n (number of experiments) is defined in the Figure legends.

## Results

### OMI/HtrA2 binds via the PDZ-domain to the NG2 proteoglycan

**Fig 1:**
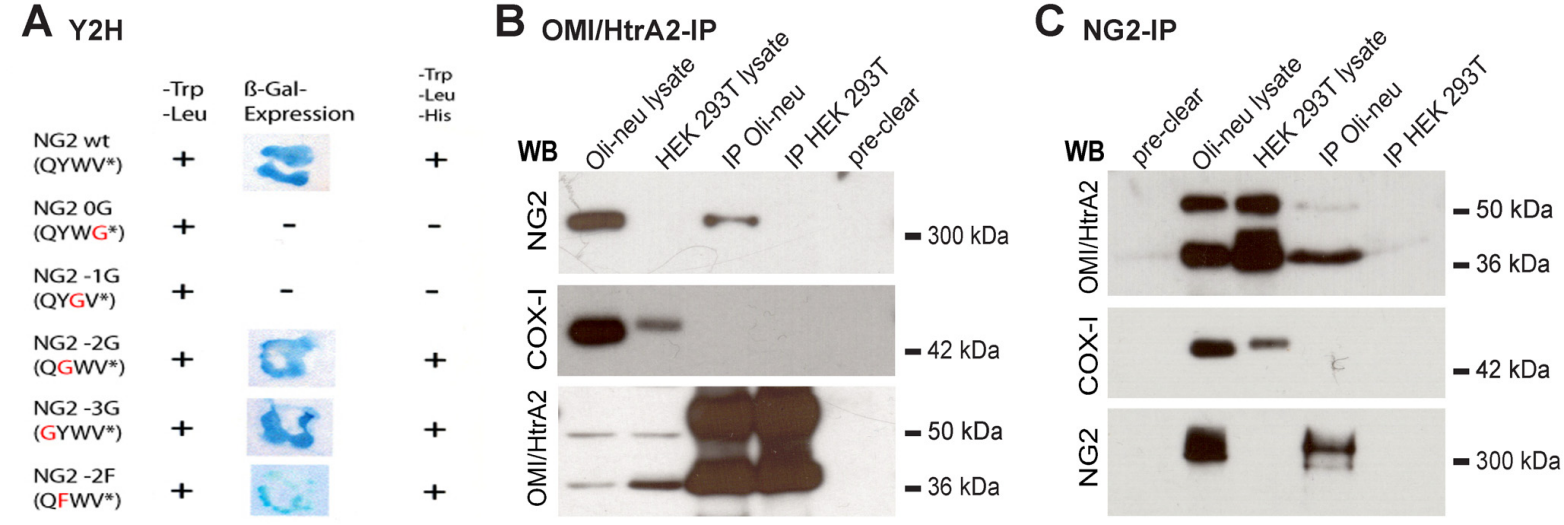
Binding of the NG2 C-terminus to the PDZ-domain containing protein OMI/HtrA2. **A**) Yeast two hybrid screen: Yeast cells were transformed with the serine protease OMI/HtrA2 and positive clones defined by ß-Gal-expression. Selection was on Trp-, Leu- and His-deficient media. The NG2 PDZ-binding motif and different mutants (red letters) of this domain were tested for binding. **B)** Lysates of Oli-neu cells and HEK-293T cells (as a control) were immunoprecipitated with pcOMI/HtrA2 antibody and immunoblotted as indicated. Endogenous NG2 is coimmunoprecipitated with the OMI/HtrA2-IP. As a negative control, the mitochondrial membrane protein COX-1 was analysed. **C)** Lysates of Oli-neu cells and HEK-293T cells were immunoprecipitated with monoclonal NG2 antibody and immunoblotted as indicated. Endogenous OMI/Htra2, mainly the processed 37 kDa form, is coimmunoprecipitated with the immunoprecipitated NG2-IP. As a negative control, the mitochondrial membrane protein COX-1 was analysed. In the pre-clear fraction, beads without the antibodies served as additional controls.

To identify the interaction partners of NG2, the complete NG2 C-terminus was used as bait in a Yeast two hybrid (Y2H) screen as previously described [27]. In this screen, two independent library plasmids represented fragments of murine OMI/HtrA2, a PDZ domain protein. To compare the PDZ-binding-motif of NG2 with the published data for sequences known to bind to OMI/HtrA2, different NG2 mutant constructs were designed. The amino acids from position 0 to -3 were replaced with glycine. Fig 1A shows that the amino acids at position 0 and -1 are essential for the interaction. Positions -2 and -3 are not essential as binding still occurs when they are substituted with glycine.

The binding between NG2 and OMI/HtrA2 protein observed in the Y2H screen was confirmed by immunoprecipitation (IP). IP using pcOMI antibody from Oli-neu lysates, an OPC cell line [28] and blotting the IP with monoclonal antibody recognising NG2, shows that NG2 is coimmunoprecipitated (Fig 1B lane 3). As a negative control, the OMI-IP was performed from HEK293T cells, which lack endogenous NG2 expression. As expected, no NG2 signal is observed in the OMI-IP on the WB (Fig 1B). As an additional negative control, we analysed the OMI-IP with antibodies to COX-I, a mitochondrial protein that like OMI is localised in a complex in the IMS. No COX-I signal is observed in the OMI-IP.

A monoclonal antibody against NG2 [25] was used to IP Oli-neu lysates. The precursor (50 kDa) and the processed forms (36 kDa) of OMI/HtrA2 are detectable in the lysates and in the IP although the cleaved form of OMI appears to be preferentially precipitated (Fig 1C). Again, WB using antibodies to COX-1 showed the specificity of the IP and the negative control with NG2-negative HEK293T lysates showed no signal. In the pre-clear lanes the beads were incubated alone with the lysates, to exclude unspecific binding of NG2 or OMI to the beads.

Together with the Y2H analysis, these results demonstrate that OMI/HtrA2 can bind to the NG2 proteoglycan.

### OMI/HtrA2 is translocated into the cytosol and binds to the NG2 proteoglycan in stressed Oli-neu cells

**Fig 2:**
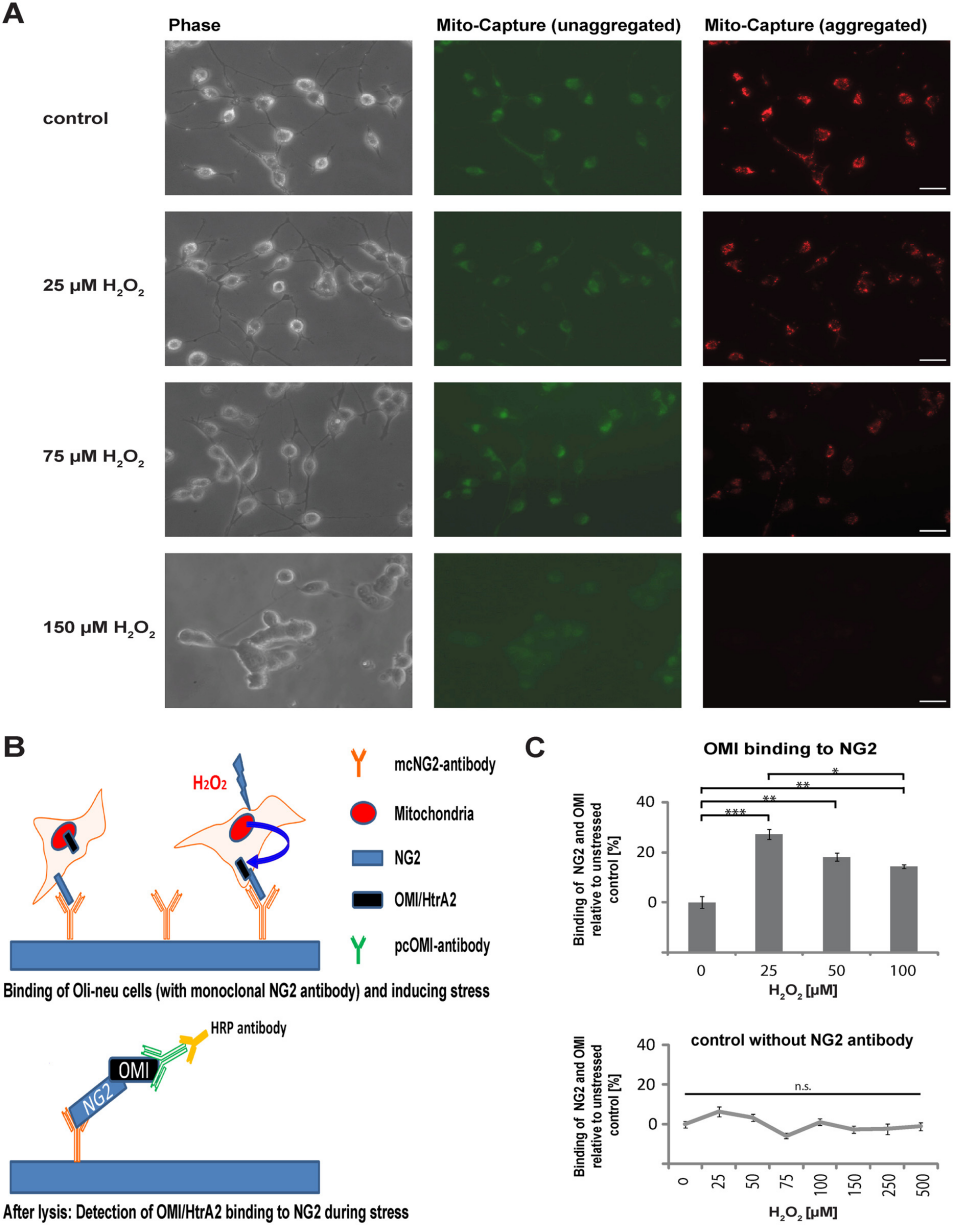
OMI/HtrA2 binds to NG2 in Oli-neu cells subjected to oxidative stress. **A)** Mito-Capture dye fluoresces green in the cytosol. In intact mitochondria with normal membrane potentials the dye fluoresces red. With increasing concentrations of H_2_O_2_ for four hours resulting in oxidative stress, the mitochondrial membrane potential is disrupted and the red fluorescence is diminished. **B)** Scheme of the CoIP ELISA: living cells were bound to plate with the monoclonal NG2 antibody and then cultured for five hours in the presence of H_2_O_2_. The cells were then lysed and OMI/HtrA2 that was released from mitochondria into the cytosol and bound to the NG2 proteoglycan was detected with polyclonal OMI/HtrA2 antibody and horseradish peroxidase-coupled anti-rabbit antibody. **C)** Binding of OMI/HtrA2 to NG2 is detected at different concentrations of hydrogen peroxide and compared to unstressed control cells, which are set as 0%. Pooled data from three experiments are shown (n = 3, SEM, unpaired Student’s t-test: p-values ***<0.001; **<0.01; *<0.05). In controls lacking plate-bound NG2 antibody, increased levels of oxidative stress do not significantly alter the background signal detected with the OMI and HRP-coupled antibodies.

Having shown that OMI/HtrA2 and NG2 can interact in a cell lysate prepared with detergent, we wanted to test if this interaction can be detected in a more physiological assay. We used the fluorescent dye Mito-Capture to analyse damage to the mitochondrial membrane under stress. When the mitochondrial membrane potential is intact the dye aggregates and fluoresces red. A reduction in red staining indicates disruption of the mitochondrial membrane potential, when the membrane is permeabilised for example during induction of apoptosis or cell damage. As shown in Fig 2A, the amount of red dye is reduced in cells exposed from 25 μM H_2_O_2_ to 75 μM H_2_O_2_ compared to the unstressed controls, however heterogeneity in the reaction of individual mitochondria in a cell is apparent. With 150 μM H_2_O_2_ the red staining disappears in all cells. We analysed whether in such stressed cells an increased binding of OMI/HtrA2 (released from mitochondria) and NG2 can be detected. We designed a, CoIP-ELISA”(Fig 2B) where living Oli-neu cells are bound to plates with the NG2 monoclonal antibody, which recognizes an extracellular epitope of the protein exposed on living cells. The cells were subject to stress and released OMI/HtrA2 captured by the NG2 protein then analysed by ELISA with antibodies to OMI. With H_2_O_2_ concentrations in a range from 25–100 μM, we detected a significant increase of OMI/HtrA2 that is bound to NG2 during the incubation under stress (Fig 2C) compared to unstressed control cells. At higher concentrations of H_2_O_2_ the amount of bound OMI/HtrA2 again decreases, this is likely due to increased cell death and dissolution of the cells as also suggested by the Mito Capture images. As an additional control, we performed assays with plates lacking bound NG2 antibody. The background signal measured here remained constant over a range of 25μM to 500 μM of H_2_O_2_ (Fig 2C).

### Knockdown of NG2 in Oli-neu cells results in a decrease in cell viability and increased apoptosis under oxidative stress

**Fig 3:**
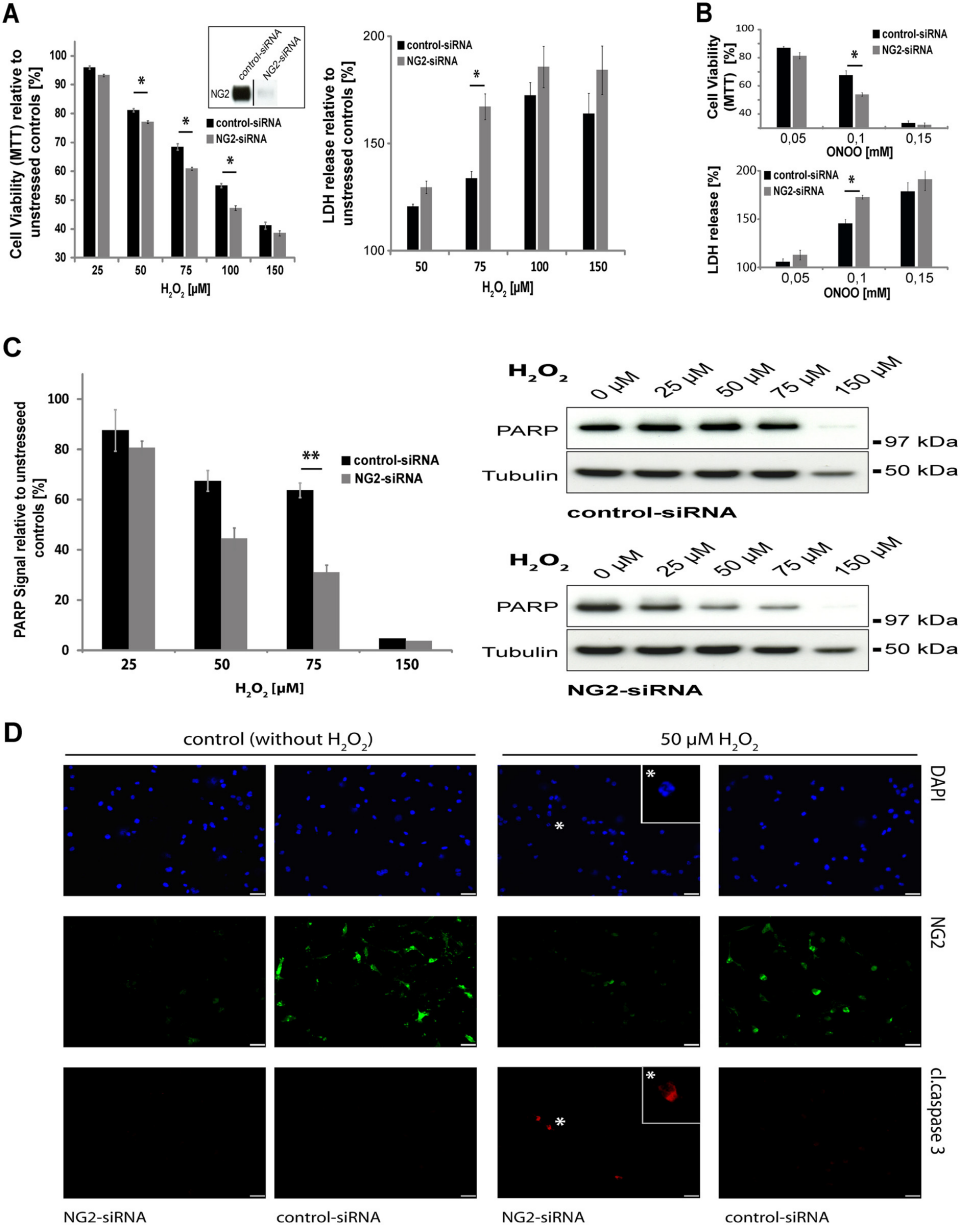
Knockdown of NG2 by siRNA results in decreased viability and a reduction in PARP levels. **A)** MTT-assay and LDH-assay with Oli-neu cells transfected with NG2siRNA and control siRNA and stressed with H_2_O_2_. MTT conversion is displayed in comparison to unstressed controls, which are set as 100% (n = 7, SEM, unpaired Student’s t-test: p-values **<0.01; *<0.05) are shown. The knockdown efficiency is shown by Western Blots for NG2 (insert). Cells are stressed for 4h. LDH release is displayed in comparison to unstressed controls, which are set as 100% (n = 4, SEM, unpaired Student’s t-test: p-values *<0.05) **B)** MTT-assay and LDH-assay with Oli-neu cells transfected with NG2siRNA and control siRNA and stressed with ONOO^-^ (peroxynitrite). MTT conversion and LDH release is displayed in comparison to unstressed controls, which are set as 100% (n = 3, SEM, unpaired Student’s t-test: p-values *<0.05) are shown. **C)** Western Blot analysis of PARP levels normalised against Tubulin in cells with and without NG2 knockdown relative to unstressed controls which are set as 100% (n = 3, SEM, unpaired Student’s t-Test: p-value **<0.01). Western Blots from one single experiment are shown as an example. **D)** NG2siRNA and control siRNA transfected Oli-neu cells stressed with 50μM H_2_O_2_ in comparison to unstressed controls. Triple staining with DAPI (*blue*), cleaved-caspase-3 (*red*) and monoclonal antibody recognising NG2 (*green*): the enlarged inserts show a cleaved-caspase-3-positive cell with a pyknotic nucleus (DAPI staining). Scale bar = 50μm.

To analyse cell viability we used the MTT-assay, which detects metabolic activity. Our experiments showed that in the OPC cell line Oli-neu, the knockdown of NG2 by siRNA induces a reduction in viability compared to the cells treated with control siRNA (Fig 3A left panel), when the cells are subjected to oxidative stress for 4 hours. The knockdown of NG2 was very efficient (Fig 3A insert). In the range between 50μM and 100μM H_2_O_2_ the reduction in viability is small but significant. As an additional measure of cell damage leading to cell death, we compared the release of the enzyme lactate dehydrogenase (LDH) [33] between cells treated with control or NG2 siRNA (Fig 3A right panel). At 75mM H_2_O_2_ cells exposed to NG2 siRNA released significantly more LDH than cells treated with control siRNA. Similar observations were made when cells were exposed to another inducer of oxidative stress: peroxynitrite (ONOO-), where again cells subjected to NG2 knockdown released more LDH and were less viable as detected by the MTT-assay (Fig 3B). Knockdown of NG2 *in vitro* has no effect on the division of the cells (S1 Fig) and the stress is applied for a relatively short time. Alterations in cell proliferation induced by NG2 knock-down are thus unlikely to be contributing to the observed differences in viability.

PARP degradation is an indicator of apoptosis [34] but also occurs in other forms of cell death e.g. necrosis [35]. Quantification of undegraded PARP (standardised against Tubulin) showed differences between cells with knockdown of NG2 and those treated with control siRNA. Exposure to 75μM H_2_O_2_ resulted in significant differences between the two groups (Fig 3C). In some cells in which NG2 levels had been reduced with siRNA, exposure to 50 μM H_2_O_2_ resulting in staining for cleaved caspase-3. DAPI staining of these cells showed a pyknotic nucleus, also a sign of apoptosis (Fig 3D *). In contrast, unstressed control cells (control siRNA) showed no activation of caspase-3 under these conditions.

### Lack of NG2 in primary OPC in mixed cultures results in an increased sensitivity to stress which is abrogated with inhibition of the OMI protease

**Fig 4:**
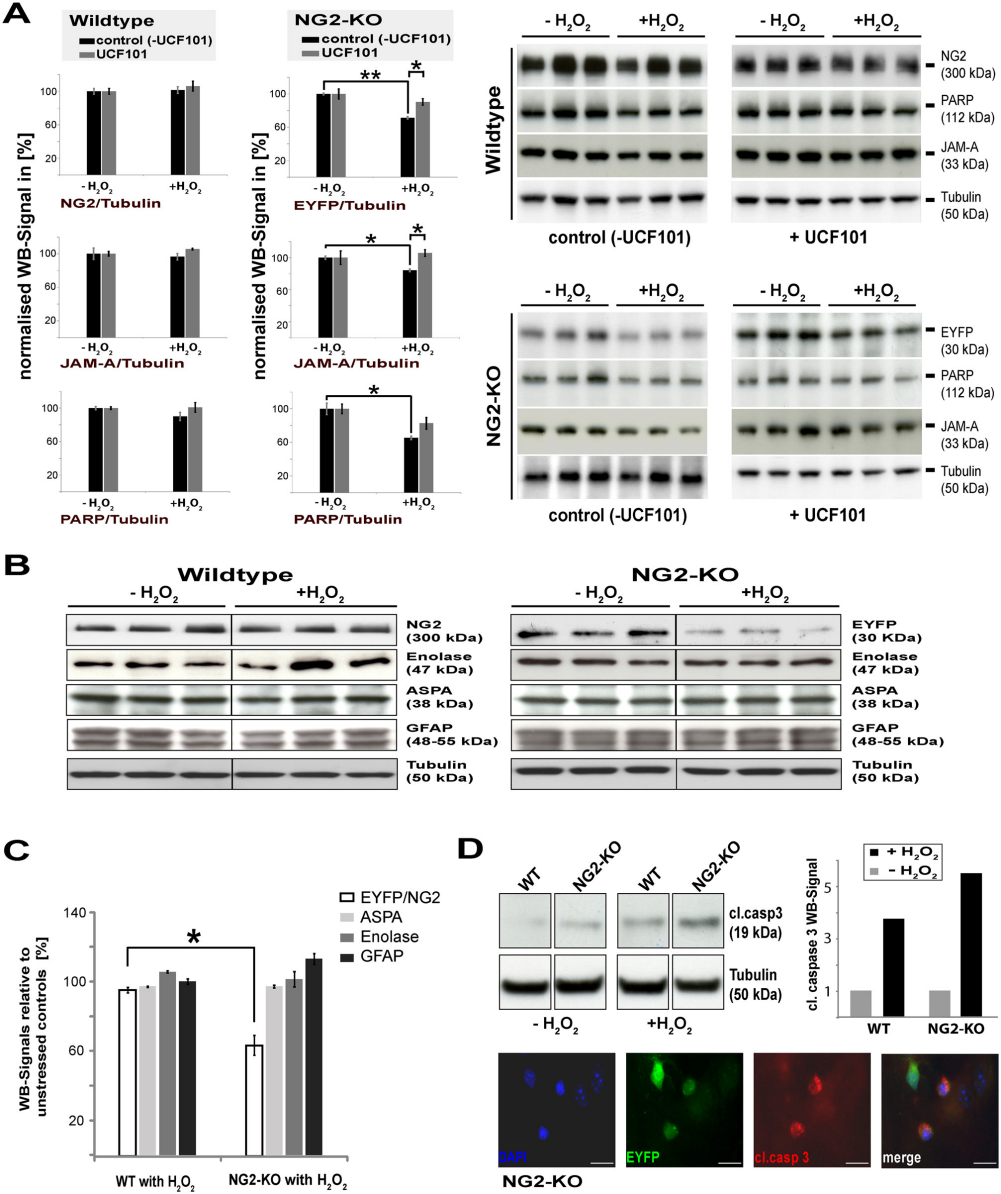
OPC lacking NG2 expression in cerebellar cultures from NG2 knockout mice are more susceptible to oxidative stress induced cell death, which depends on the OMI protease activity. **A)** Quantification of Western Blot analysis of lysates of cerebellum cultures containing all cell types of WT and NG2-KO mice with the specific OMI-inhibitor UCF101 (grey columns) or without (black columns). Cultures were exposed to H_2_O_2_ for 18 hours. OPC were quantified by blotting for NG2 (WT), EYFP (KO) or JAM-A (for WT and KO). Degradation of PARP is an indication of cell death. In the presence of the OMI inhibitor UCF101, less death of NG2-lacking (NG2-KO) OPC is observed. The WB signals of samples from six individual animals (3WT; 3NG2-KO) with the indicated antibodies are shown on the right from which the quantitative data (bar graphs) is derived. All signals are normalised to Tubulin and compared to the unstressed controls (set as 100%) (n = 3, SEM, unpaired Student’s t-Test: p-value **<0.01; *<0.05). **B)** Cerebellum cultures of wildtype (WT) mice and NG2/EYFP knockout mice (NG2-KO) were incubated with 800μm H_2_O_2_ for 18 hours, lysed and compared to unstressed controls by WB with cell-type-specific markers. Blots are shown of one experiment with three replicates. **C)** Quantification of WB signals for different cell-type specific markers against Tubulin of the cerebellum cultures, compared to unstressed cultures (EYFP and NG2 respectively for OPC, ASPA for oligodendrocytes, δ-Enolase for neurons and GFAP for astrocytes; (n = 4, SEM, unpaired Student’s t-Test: p-value *<0.05) as shown in the example (B). **D)** WB analyses with quantification of activated caspase-3 (cleaved caspase-3) of WT and NG2-KO cerebellum cultures with and without oxidative stress for 5 hours (n = 1). An example of apoptotic cell death in OPC in the cerebellum cultures from the NG2-KO mice after 5 hours of stress with 800μm H_2_O_2_ stained with cleaved-caspase-3 (red), EYFP (green) and DAPI (blue) is shown. Scale bar = 50μm.

To test the response of primary OPC to stress in a more physiological situation including the presence of other cell types, we analysed cerebellar cultures from young mice, which contain all major neural cell types. We have generated mice in which EYFP is inserted directly after the endogenous NG2 promoter [29,36]. In the homozygous state, these mice are lacking expression of NG2 (NG2-KO). We set up cerebellar cultures from NG2-KO mice and from WT animals. This allowed us to compare the response of the OPC lacking NG2 to those of wild-type animals, but also to analyse the cellular specificity of the response by examining the other cell types in the cultures. We used higher concentrations of H_2_O_2_ (800μM) because astrocytes in these mixed cultures can eliminate hydrogen peroxide very fast from the media [37]. Western Blot analysis (Fig 4A and 4B) reveals that OPC in cerebellum cultures from NG2-KO mice are more sensitive to H_2_O_2_-induced stress than OPC in cultures from WT mice. The EYFP signal of OPC lacking NG2 is reduced by about 30% during the 18 hours incubation (Fig 4A top right panel, black bars), whereas the NG2 signal of WT OPC (Fig 4A top left panel black bars) is relatively unchanged. As an independent assessment of OPC death, we analysed the expression of the OPC surface protein JAM-A [38] (Fig 4A middle panels), which is equally expressed in both WT and NG2-KO OPC. This yielded comparable results, again showing loss of NG2-KO OPC in response to stress. To analyse whether the enhanced cell death in the NG2-KO cultures is OMI/HtrA2-dependent, we used UCF101, which is considered a specific inhibitor of the OMI-protease, although UCF101 has been reported to exhibit some proapoptotic side effects [39]. In cultures from NG2-KO mice incubated with UCF101, far fewer damaged OPC are observed under stress, indicated by the stronger EYFP and JAM-A signal, suggesting that the cell death is largely OMI/HtrA2-dependent (Fig 4A top and middle right-hand panels, grey bars). Additionally, less PARP is degraded (Fig 4A bottom panels), confirming the reduction in cell death. In WT cultures we observed a slight but non-significant degradation of PARP in response to stress, which was absent in the presence of UCF101. Similarly, the reduction in JAM-A expression in NG2-KO OPC under stress was largely abrogated in the presence of UCF101. In contrast to the increased sensitivity of OPC lacking NG2 to stress, other cell types in the cultures from either WT or NG2-KO mice (mature oligodendrocytes: ASPA, neurons: γ-enolase, astrocytes: GFAP) display no noticeable cell death under these stress conditions (Fig 4B and 4C).

To determine whether apoptosis is likely to be contributing to the reduction in the EYFP or JAM-A signal and thus OPC number, cerebellum cultures from NG2-KO and wildtype mice were stressed for 5 hours and activation of the apoptosis-associated enzyme caspase-3 analysed by blotting for the caspase-3 cleavage product. WB analysis shows that exposure to oxidative stress results in a stronger signal for cleaved caspase-3 in NG2-KO cultures (Fig 4D) compared to WT cultures. Furthermore, in cultures from NG2-KO mice some OPC (recognised by expression of GFP) were stained by antibodies recognising cleaved caspase-3 and also exhibited pyknotic nuclei, as evidenced by DAPI staining (Fig 4D). In wildtype cultures, cleaved-caspase-3 positive OPC were only seldom observed under stress.

### OMI/HtrA2 protease activity is reduced by binding the NG2 PDZ-binding motif

**Fig 5:**
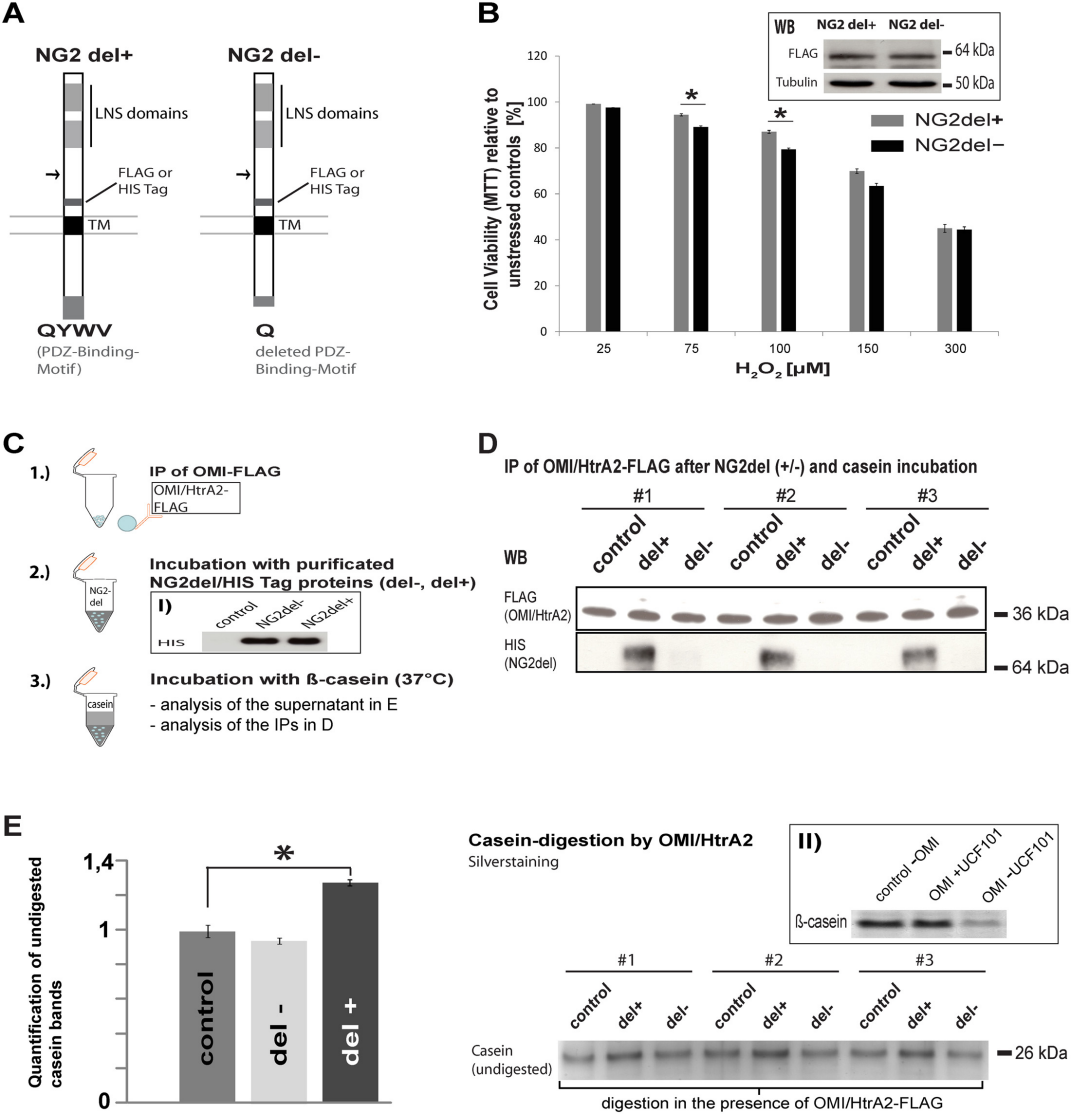
The NG2 PDZ-binding motif contributes to the protective function by reducing the protease activity of OMI/HtrA2. **A)** Diagram showing the domain organization of the NG2del proteins. The FLAG Tag was used in transfected cells in the MTT-assay, the HIS-Tag was utilised for purification of NG2del proteins for the protease-assay. TM indicates the transmembrane domain; the *arrow* indicates the position of the deletion (amino acids 478–2164) in NG2del. The amino acids QYWV represent the PDZ binding motif in the NG2del+ construct. **B)** MTT-assay of HEK293T cells transfected with an NG2-FLAG construct with an intact PDZ-binding-motif, which interacts with OMI/HtrA2 (NG2del+) and a NG2 construct in which the PDZ-binding-motif was deleted (NG2del-). The reduced MTT conversion with stress for 4h is compared to unstressed controls. HEK293T cells that express NG2del+ showed a higher percentage of viable cells under oxidative stress (n = 3, SEM, paired Student’s t-Test: p-value *<0.05). WB analysis with FLAG antibody (insert) shows similar expression of each transfected construct using the same amount of cells and plasmids. **C)** Scheme of the protease assay: to test the influence of NG2 on the OMI/HtrA2 protease activity, purified NG2del-HIS proteins (NG2del- without PDZ-binding motif and NG2del+ with the motif) were incubated with OMI/HtrA2-FLAG immunoprecipitates, obtained from HEK cells transfected with FLAG-tagged OMI. These were then incubated with the OMI/HtrA2 substrate β-casein for 5h at 37°C, to analyse whether binding of NG2 influenced the activity of the OMI serine protease. WB analysis (panel I) shows that equal amounts of the NG2del fusion proteins are used for each assay, as a control untransfected HEK lysate was used. **D)** WB analysis of the IPs used for the casein digestion shows that equal amounts of FLAG-tagged OMI/HtrA2 are used for each assay and that only NG2del+ (with the intact PDZ-binding motif) is co-immunoprecipitated in the OMI/HtrA2-FLAG IP. **E)** The degradation of β-casein in the supernatant from the same three experiments shown in D was analysed by gel electrophoresis and silver staining. Undigested casein is shown after incubation with the complex of OMI/HtrA2 and NG2 (NG2del+ or NG2del-). The buffer from the purification of the NG2del proteins was used as a control in D and E. Quantification of the signals of the casein band (NG2del+ and NG2del-) was normalized against the buffer control (left bar graphs). When the OMI/HtrA2 protease is inhibited by the interaction with the NG2del+ protein less casein is digested resulting in stronger signals (n = 3, SEM, unpaired Student’s t-Test: p-value *<0.05). In panel II it is shown that the OMI/HtrA2 protease inhibitor UCF101 largely prevents digestion of β-casein in this assay.

To further analyse how the interaction between NG2 and OMI/HtrA2 contributes to the enhanced viability of NG2-expressing cells, we transfected NG2-lacking HEK293T cells with an NG2 construct (Fig 5A), in which the PDZ-binding-motif that binds to OMI/HtrA2 is present (NG2del+), and as a control a construct lacking the PDZ-binding-motif (NG2del-). We generated 4 different constructs, in each case NG2del+ and NG2del- had either a FLAG or a HIS Tag inserted near the transmembrane domain. MTT-assays showed that HEK cells expressing the NG2del-FLAG protein with the intact PDZ motif (NG2del+) exhibited an enhanced viability after oxidative stress, compared to cells expressing NG2del-. A significant difference between NG2del+ and NG2del- was observed with 75 to 100 μMH_2_O_2_ (Fig 5B). To test if this interaction with NG2 influences the protease activity of OMI/HtrA2, we designed a protease assay with the known OMI/HtrA2 substrate β-casein (scheme in Fig 5C). We incubated IPs of FLAG-tagged OMI derived from HEK cells transfected with FLAG-tagged OMI/HtrA2 together with NG2del+ or NG2del- proteins isolated using nickel affinity columns from HEK cells transfected with HIS-tagged NG2del constructs. The resulting complexes were then incubated with a defined amount of ß-casein. In Fig 5D can be seen that only the NG2del+ protein binds OMI/HtrA2. Inclusion of the OMI inhibitor UCF101 results in no change in the casein bands between samples with and without the OMI protease (Fig 5E; insert II). Only the complex with the NG2del+ protein with the PDZ-binding-motif (del+) results in a stronger band of ß-casein (decreased digestion) as shown in Fig 5E.

### In human glioblastoma, NG2 protects against apoptosis induced by oxidative stress

**Fig 6:**
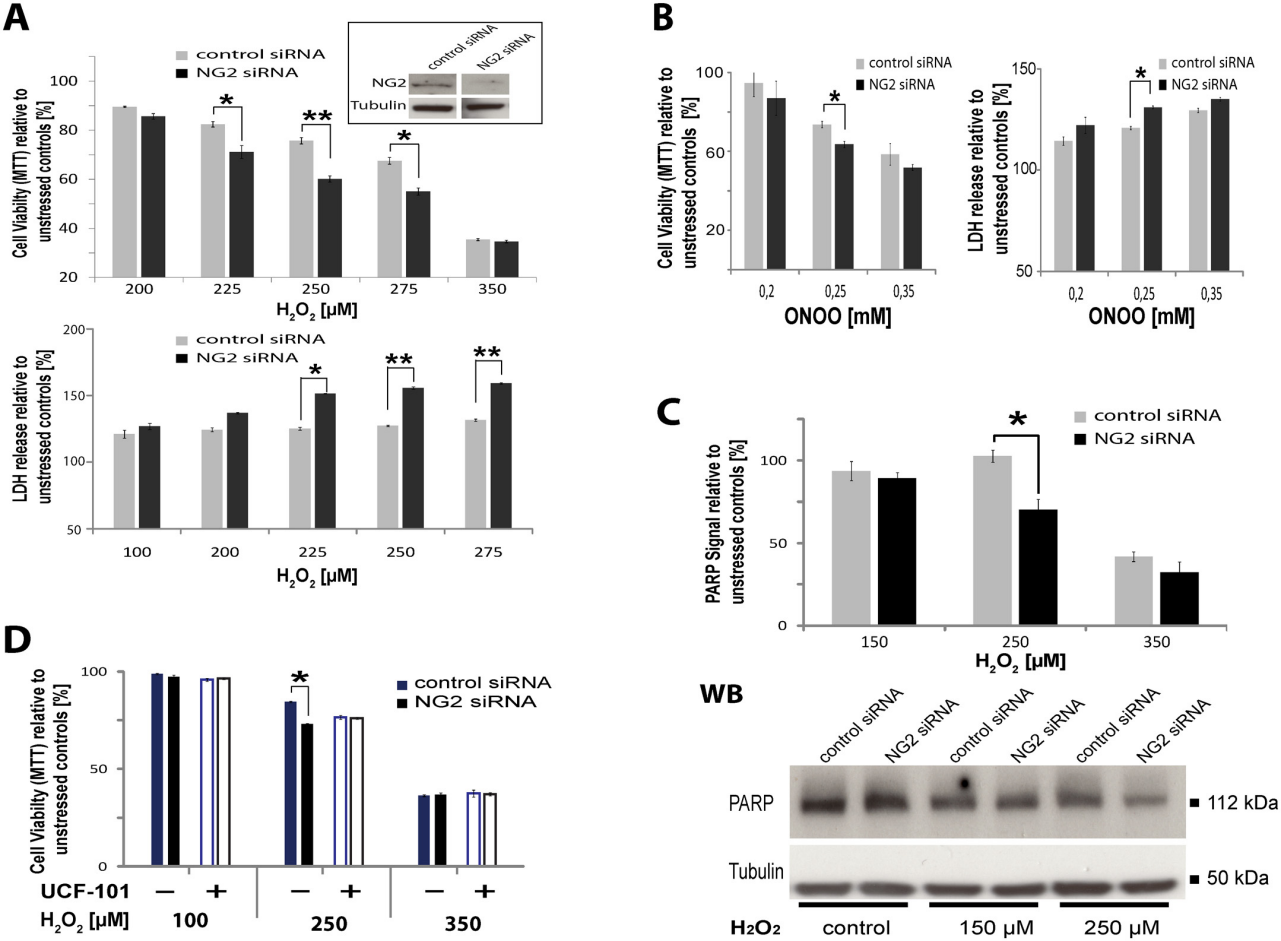
Expression of NG2 by glioblastoma confers resistance to induction of cell death with oxidative stress dependent on OMI protease activity. **A)** MTT-assay with NG2-positive GBM cells (line R10) transfected with NG2siRNA or control siRNA. MTT conversion is displayed in comparison to unstressed controls, which are set as 100% (n = 4, SEM, unpaired Student’s t-Test: p-values **<0.01; *<0.05). LDH release is displayed in comparison to unstressed control (100%) (three replicates, SEM, unpaired Student’s t-Test: p-values **<0.01; *<0.05). WB analysis of the efficiency of NG2 knockdown in GBM cells is shown in the insert. **B)** MTT-assay and LDH-assay with GBM cells (R10) transfected with NG2siRNA and control siRNA and stressed with ONOO^-^ (peroxynitrite). MTT conversion and LDH release are displayed in comparison to unstressed controls, which are set as 100% (n = 3, SEM, unpaired Student’s t-test: p-values *<0.05) are shown. **C)** Western-Blot analysis of PARP levels normalised against Tubulin in cells with and without NG2 knockdown. PARP levels of unstressed controls are set as 100% (n = 4, SEM, Student’s t-Test: p-value *<0.05) and an example of WB-analysis of PARP levels from one single experiment is shown. **D)** MTT-assay with NG2-positive GBM cells transfected with NG2siRNA or control siRNA and incubated with or without the OMI inhibitor UCF101. MTT conversion is displayed in comparison to unstressed controls, which are set as 100% (4 replicates, SEM, unpaired Student’s t-Test: p-values *<0.05).

A classic feature of human glioma cells surviving radio- and chemotherapy in patients is the expression of NG2 [12]. Reduction of NG2 protein levels in glioblastoma cells expressing high levels of NG2 (R10) by siRNA, resulted in a decrease of cell survival under oxidative stress (225–275 μM H_2_O_2_) indicated by a lower signal in the MTT-assay (Fig 6A) and lower levels of undegraded PARP (Fig 6C), indicating an increased cell death compared to cells treated with control siRNA. Measurement of LDH release from dying cells (Fig 6A) confirmed these results. Similar results were observed when we used peroxynitrite (ONOO-), another inducer of oxidative stress, where again cells subjected to NG2 knockdown released more LDH and were less viable as detected by the MTT-assay (Fig 6B). When the OMI protease is inhibited with UCF101, the difference in viability between control and NG2 siRNA treated cells disappears (Fig 6D), showing that the protective function of NG2 is dependent on OMI protease activity, similar to the effects observed in OPC.

## Discussion

### NG2 binds to OMI/HtrA2 via the PDZ binding domain under oxidative stress

We show by Y2H and biochemical analysis that the well-established PDZ-binding motif of NG2 binds to the PDZ domain of OMI/HtrA2. For binding to OMI/HtrA2, the amino acids at the C-terminus of the PDZ binding motif must be hydrophobic and Valine was most prevalent at the C-terminal position 0 and Tryptophan was dominant at Position -1 [40]. The PDZ-binding-motif of NG2 consists of amino acids_-3_QYWV_0_, the hydrophobic character on position 0 and -1 is given. We confirmed the Y2H binding with biochemical analysis of cell lysates. These results add OMI/HtrA2 to the list of PDZ-domain proteins that can bind the C-Terminus of NG2. These to date comprise GRIP1 [27], Syntenin [26], MUPP1 [41] and OMI/HtrA2.

Since OMI/HtrA2 is a protein normally found in the mitochondrial intermembrane space and NG2 is a membrane protein, these molecules would not meet in the same subcellular compartment under normal physiological conditions. Under conditions of cell stress where mitochondria membranes are permeable, OMI/HtrA2 is released into the cytosol where it can activate apoptosis by binding and degradation of the IAPs [21]. Our experiments show that OMI binds to the cytoplasmic PDZ binding motif of NG2 in living cells when the mitochondrial membrane system is permeabilised by oxidative stress.

The NG2 ectodomain can be isolated from the CNS in the absence of detergent [42] and we have shown recently that the ectodomain cleavage is under control of neuronal activity and mediated by the alpha secretase ADAM 10 [36,43]. A range of proteases can cleave NG2 and there are reports that NG2 protein levels increase in CNS lesions [44]. However, the dominant form of NG2 present in cells in culture is the full length NG2 protein. Expression of a truncated form of NG2 in cells *in vitro* (NG2 del+) also resulted in increased cell viability compared to NG2 del- constructs lacking the PDZ-binding domain. Interestingly, expression of NG2 del constructs which are processed by the same enzymes as full length protein, leads to very high levels of the NG2 CTF [36,45]. Binding of OMI to the NG2 CTF via the PDZ-binding domain is thus implied. Furthermore it is very unlikely that the cleavage of NG2 will have a major effect on the anti-apoptotic properties of the protein.

### OPC lacking NG2 are more susceptible to OMI protease-dependent cell death induction than wildtype OPC

The resistance of OPC to stress is thought to be due to protective pathways such as glutathione, but additional factors such as the expression level of procaspase-3, or the ratio of pro- and anti-apoptotic molecules undoubtedly contribute [46–48]. As released OMI/HtrA2 triggers apoptosis, we assumed that the interaction with NG2 could have an influence on cell survival. Reduction of NG2 by siRNA in an OPC cell line as well as OPC lacking NG2 in a more physiological system, a cerebellar culture system containing all neural cell types, showed that the NG2 protein protects OPC against induction of cell death induced by oxidative stress. Astrocytes can protect other CNS cells by supplying them with precursor molecules of the antioxidant glutathione [49] and thus the results with primary cultures are instructive. An increased signal for cleaved-caspase-3 and an increased degradation of the DNA repair enzyme PARP suggested that some of the cultured cells may be dying by apoptosis, although we were unable to detect the 85kDa form of PARP in OPC. A previous study indicates that NG2 can be an anoikis receptor [50], thus promoting rather than inhibiting death in this case. These experiments are performed in fibroblasts and we assume that NG2 may play different roles in different cell types under different conditions.

OMI stimulates apoptosis by binding and degrading the IAPs. We demonstrated that the increased cell death in OPC from NG2-KO animals is dependent on the OMI protease as a specific inhibitor of the OMI protease reduced the amount of cell death seen in the mixed cerebellar cultures. Analysis of the cleavage of β-casein, a substrate of OMI/HtrA2, demonstrated that NG2 binding reduced the OMI protease activity. In contrast, NG2 itself is not a substrate of the OMI protease. Regulatory effects on the OMI protease have been shown for other PDZ proteins, which bind to OMI/HtrA2: these include presenilin and the large tumour suppressor kinase LATS1/WARTS [51,52]. In the case of NG2, the reduced protease activity of OMI/HtrA2 is likely to have an influence on the activation of apoptosis. It is also possible that the interaction with NG2 prevents binding of activators of the OMI/HtrA2 protease, thus further leading to a reduction in cell death. In *D*. *Melanogaster* it has been reported that DIAP1 (Drosophila inhibitor of apoptosis proteins 1) is not only degraded by OMI but also mediates polyubiquitination of OMI for proteasomal degradation. This protects cells from lethal effects of OMI/HtrA2 in the cytosol [53], especially in conditions when only a small amount of OMI/HtrA2 is translocated into the cytosol by oxidative damage of a few mitochondria.

### In glioblastoma cells, NG2 exerts a protective function under oxidative stress conditions dependent on OMI protease activity

Studies showed that the NG2 proteoglycan promotes resistance of glioblastoma cells to apoptosis-inducing reagents [54]. Our work shown here demonstrates that the expression of NG2 by glioblastoma protects against cell damage and cell death triggered by oxidative stress. The increased resistance of glioma cells expressing NG2 to chemo- and radiation therapy has been attributed to the cis-interaction of NG2 with α3β1-Integrin (via the ß-1 subunit) which activates the PI3K/Akt signal pathway [12]. We observed slight but non-significant differences in phosphorylation of Akt between NG2 siRNA and control siRNA treated glioma cells under stress conditions where NG2 expression confers better survival (data not shown), but we observed that in GBM cells treated with the specific OMI inhibitor UCF the protective effect of NG2 is eliminated. We thus conclude that the activation of the PI3K/Akt pathway through NG2 is not solely responsible for increased cell survival but that expression of NG2 is additionally protective via other mechanisms. Our experiments with NG2 constructs with and without PDZ-binding motifs, further show that the intracellular NG2 PDZ-binding motif (which does not itself interact with integrins), is also contributing to stress resistance.

### NG2 binding to the OMI/HtrA2 may protect OPC and NG2-expressing glioma cells from oxidative stress in humans

Our work suggests that the expression of NG2 counteracts the extreme sensibility of OPC to damage and induction of cell death as is seen in white matter injury in premature infants and lesions in Multiple Sclerosis [3–6]. OPC divide extensively during development but also in the adult brain: 5–8% of total cells are NG2+ OPC with mitotic and migratory potential [55] which may be one reason why these are often the cells of origin for glioma [10,11]. Expression of NG2 promotes migration and polarity development [14], properties important for glioma dissemination. The observations that aggressive glioblastoma often strongly expresses NG2, that radio- and chemoresistance is promoted by NG2 [54] and that NG2+ cells sorted from tumour material have an especially aggressive signature [8] match our findings that NG2 expression is furthermore stress-protective. Our data shown here suggest that the PDZ-binding motif of the NG2 protein can help to protect against damage and cell death in response to stress by sequestering and reducing the protease activity of the apoptosis-inducing serine protease, OMI/HtrA2.

## Supporting Information

S1 Fig OPC proliferation after NG2 knock-down. The percentage of proliferating Oli-neu cells (Ki67+/DAPI+) were determined after 24h and 48h of culture, as shown by the immunofluorescent pictures in **A** (24h). **B)** Stable NG2 knock-down lines (shNG2) were compared to control (shLuc) lines and showed no significant differences in cell division. At least 500 cells were counted for each condition and time point from two independent experiments. (Scale bar = 40 μm.)
(TIF)
