# Supplementary figures and images for: The NG2 Proteoglycan Protects Oligodendrocyte Precursor Cells against Oxidative Stress via Interaction with OMI/HtrA2
Source: PLoS One. 2015 Sep 4;10(9):e0137311. doi: 10.1371/journal.pone.0137311 (PMC4560422; doi:10.1371/journal.pone.0137311)

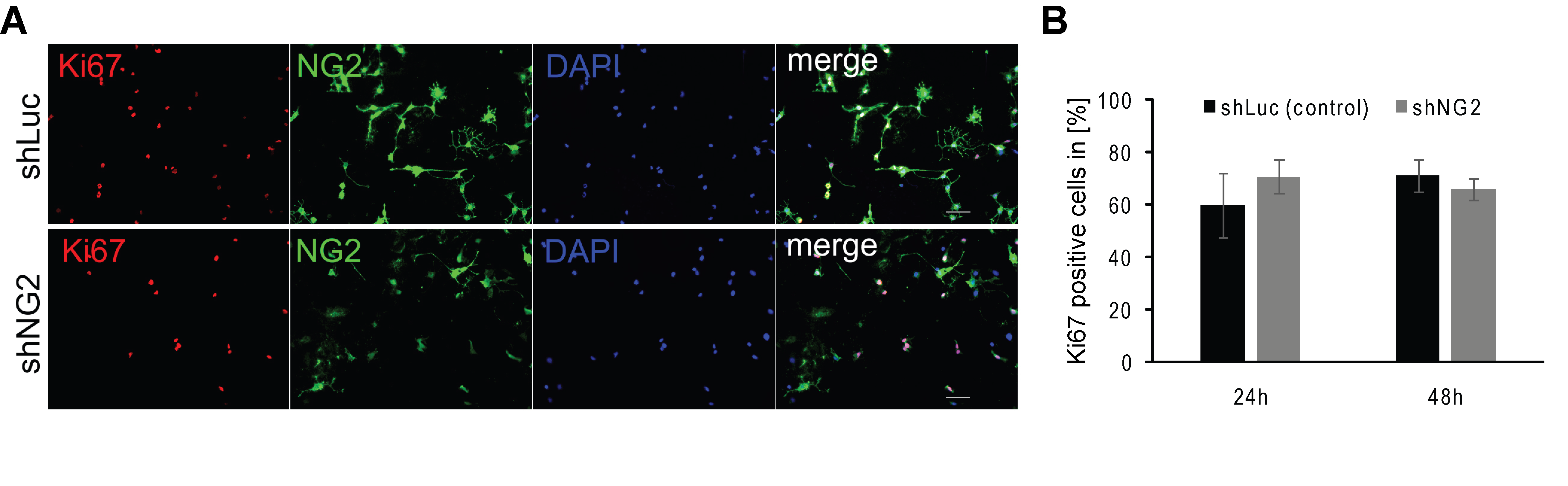

Supplement: S1 Fig — The percentage of proliferating Oli-neu cells (Ki67+/DAPI+) were determined after 24h and 48h of culture, as shown by the immunofluorescent pictures in A (24h). B) Stable NG2 knock-down lines (shNG2) were compared to control (shLuc) lines and showed no significant differences in cell division. At least 500 cells were counted for each condition and time point from two independent experiments. (Scale bar = 40 μm.) (TIF) [file pone.0137311.s001.tif]
